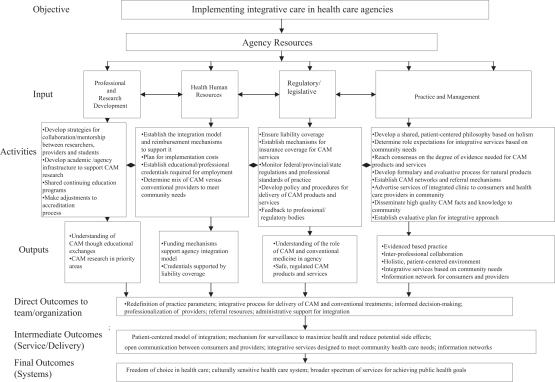# A Model for Implementing Integrative Practice in Health Care Agencies

**Published:** 2008-02-14

**Authors:** Chris Patterson, Heather M. Arthur

**Affiliations:** 1 McMaster University, School of Nursing, Rm 2J21b, 1200 Main St. West, Hamilton, ON, L8N 3Z5; 2 Heart and Stroke Foundation of Ontario Chair in Cardiovascular Nursing Research McMaster University, Rm 2J29, 1200 Main St. West, Hamilton, ON, L8N 3Z5

**Keywords:** integrative care, integrative practice model, complementary/alternative medicine, inter-paradigm teams

## Abstract

Over the last few years, there has been increased awareness and use of complementary/alternative therapies (CAM) in many countries without the health care infrastructure to support it. The National Centre for Complementary and Alternative Medicine referred to the combining of mainstream medical therapies and CAM as integrative medicine. The creation of integrative health care teams will definitely result in redefining roles, but more importantly in a change in how services are delivered. The purpose of this paper is to describe a model of the necessary health care agency resources to support an integrative practice model. A logic model is used to depict the findings of a review of current evidence. Logic models are designed to show relationships between the goals of a program or initiative, the resources to achieve desired outputs and the activities that lead to outcomes. The four major resource categories necessary for implementing integrative care are within the domains of a) professional and research development, b) health human resource planning, c) regulation and legislation and d) practice and management in clinical areas. It was concluded that the system outcomes from activities within these resource categories should lead to freedom of choice in health care; a culturally sensitive health care system and a broader spectrum of services for achieving public health goals.

Social, political, professional and economic forces have, and will continue to provide considerable influence on how health care professionals deliver care. As any health care discipline changes its scope of practice in response to these market forces, there will be an impact on other disciplines’ practice domains, as well as on the legislation and regulations governing professional structures. Bucher (1988) captured this “domino effect” in her statement, “What we now have is a teeming arena, with more and more groups crowding in, jockeying for position, rearranging themselves, and some even pushing at the barricades of medicine” (p. 131).

The National Centre for Complementary and Alternative Medicine (CAM) (2004) referred to the combining of mainstream medical therapies and CAM as integrative medicine. The creation of integrative health care teams will definitely result in redefining roles, but more importantly in a change in how services are delivered.

To date, the integration of CAM has been driven by factors such as a) the degree of availability of CAM, b) insurance coverage for CAM services (Mootz and Bielinski, 2001; Andrews and Boon, 2004), c) the use of CAM with conventional medical treatments by consumers (Tataryn and Verhoef, 2001; Libster, 2003) and d) the emergence of practitioners combining CAM therapies with conventional practice (Andrew and Boon, 2004). For some practitioners, integrative medicine is difficult to achieve since questions linger about which therapies should be integrated and how should they be integrated (Richardson, 2001). However, despite the chaotic nature of CAM introduction into health care systems, there continues to be a slow shift away from the traditional belief that healing can be explained solely by the bio-medical model towards a holistic approach in which many therapies could conceivably be used in prevention and treatment. Ernst et al. (1995) refer to this shift as “…diversifying the conceptual frameworks of medicine” (p. 506).

Over the last few years, there has been increased awareness and use of CAM in many countries, including Canada, without the health care infrastructure to support it (Statistic Canada, 1995; Millar, 1997; Millar, 2001). The importance of provider consensus has been highlighted in regard to the philosophical underpinnings of an integrative approach to care; equal consideration for the healing potential of differing treatment modalities; commitment to holism; and practice environments that support partnerships and dissemination of evidenced based knowledge on CAM (Francoeur et al. 2006). Although provider consensus is a necessary step in the process of advancing integrative medicine, a health systems strategy is also required in order to foster implementation. The purpose of this paper, therefore, is to describe a model of the necessary health care agency resources to support an integrative practice model.

## Methods

The following electronic data bases were searched from 1996 to 2006: CINAHL, PubMed (Medline), ERIC, PsycInfo, AMED, EMBASE, Sociological Abstracts, Ageline, Health and Psychological Inst., Health Star and Journal @ Ovid. The search strategy was refined through the iterative testing of several terms and incorporation of new terms as relevant citations were identified. The following keyword combinations were used: complementary/alternative medicine and integration; alternative medicine and integration; and complementary medicine and nursing and integration. A criterion for selection was the presence of at least one of the known key elements which define practice within health care agencies: a) professional and research development, b) health human resources, c) regulation and legislation and d) practice and management. Any other influencing factors for role development which were unique to integrative practice models were identified by one of the authors and incorporated into the search strategy. The purpose of the review was to extract from the literature activities which were unique to *implementation* of integrative practice models rather than to discuss common role development strategies. Since this area of research has most often used qualitative approaches and expert opinion, there was no attempt to exclude on the basis of design. Articles on curriculum development were not reviewed; again because they were not directly related to the clinical implementation of integrative models. The literature review only included articles in English; this may be a limitation.

Logic models are visual displays that show the relationships among resources invested, the activities performed, and the resulting outcomes in relation to desired goals. Therefore, logic models are templates to be completed, with appropriate information from reliable sources, to guide program development or an initiative within an organization. The components of a logic model applied to integrative health care practice are as follows: 1) inputs are generally viewed as what needs to be invested in order meet the desired outputs; 2) outputs are the activities which lead to outcomes; and 3) outcomes are the benefits to patients, families, health care professionals, organizations and the health care team (Taylor-Powell, Jones and Henert, 2002). The inputs of this logic model were based on known, important parameters defining practice in the health care system; important organizational considerations when implementing a practice; and what was identified in the literature review as important to implementing integrative practice. The remaining sections of the logic model- activities, outputs and outcome- were identified and extracted from the findings of the literature search.

## Findings

The logic model provides a visual representation of the findings of the literature review and addresses the purpose of this paper (Fig. 1). Logic models are designed to depict the relationships between the goals of a program or initiative, the resources (inputs) to achieve desired outputs, and the activities that lead to outcomes (Rush and Ogborne, 1991). The resources seen as necessary for successful implementation of integrative care are identified as the inputs and are attached to associated activities in the model.

## Discussion

### Health human resource planning

A unique aspect of health care planning for integrative care compared to the more traditional approach is the challenge created in coordinating “inter-paradigm” teams. This type of health care planning requires matching diverse expertise, which is based in different ways of knowing, with treatment modalities. Forecasting the “right” blend of expertise for integrative care will depend on 1) knowing professional boundaries, 2) estimating the number of available CAM practitioners based on educational training, regulatory status and professional structure, and 3) determining the demand of CAM by consumers within the geographical area served by the agency (Meenan and Vuckovic, 2003).

The agency must have a current and projected assessment of its health care needs, the role of an integrative approach in meeting them, and the cultural norms that influence beliefs about health care. A careful analysis of cultural norms is a good first indicator of potential success of integrative care within the area since the historical roots of CAM are embedded deep within the traditions of society. Internal and external stakeholders’ input into the human health care planning phase will facilitate buy-in to programs, inform decision-making on the appropriate mix of healing practices and providers, and provide an ongoing forum for evaluative purposes.

Of the four models of integration proposed by Leckridge (2004), a patient-centered model of integration as the one most likely to support the development of “integrative medicine”. This model is centered on the biopsychosocial approach to health and illness; this approach is embodied by partnerships, patient goals, safe, regulated products and services that are consistent with patient values, and elimination of boundaries between CAM and conventional health care. Tataryn and Verhoef (2001) supported Leckridge’s model of integration in their conceptualization of what is necessary for systematic integration. Systematic integration requires the use of evidenced based practice and clinical expertise; balances the needs of consumers and practitioners; commits to society and focuses on a holistic approach to health and illness. However, these broad recommendations for models of practice do not provide detail for program development or the evidence of what constitutes an optimal model of integrated CAM provisions (Haselen et al. 2004). Therefore, the biggest challenge for agencies will be in identifying an integrated model of practice that fits with the organizational culture, the health care needs of the community and the budgetary burden for implementing those services, especially if estimated costs cannot be recovered through third party reimbursement.

Researchers have reported other practice models in which: 1) CAM and conventional practitioners co-exist but are distinct; 2) conventional practitioners provide medical and CAM services; or 3) conventional practitioners refer to CAM providers (Mootz and Bielinski, 2001; Tataryn and Verhoef, 2001; Kelner et al. 2003). Each of these types of arrangements have the potential to result in dis-integration, poor quality of care, alteration in the nature and traditions of CAM and potential marginalization of CAM providers. Employers and practitioners must work with key stakeholders to discuss how they will redefine practice parameters based on the preferred model of integration and establish inter-professional boundaries within regulatory parameters. When developing their practice model, team members must give careful thought to the benefits of coordinating conventional medicine and CAM throughout the healing process versus merging the two, which puts CAM traditions at risk (Meenan and Vuckovic, 2003; Block et al. 2004).

During the planning stage, there should also be consideration of the standard of accountability required for employment. Typically, the professionalization process for different CAM groups is associated with issues of education, self-regulation and evidence for therapies (Kelner et al. 2003). Integration can only be achieved through tolerance of the professionalization process and agency guidelines for acceptable level of educational and professional credentials. Professional organizations and federal, provincial and territorial regulations could assist employers in these types of decisions.

### Practice

Philosophical underpinnings are the foundation on which practice models are built and many authors have noted that understanding different philosophical positions is important for successful integration (Pelletier et al. 1999; Mootz and Bielinski, 2001; Tataryn and Verhoef, 2001; Barrett, 2003). However, to achieve a practice model that supports freedom of choice, it must be driven by philosophical consensus. The philosophy for integrated practice will have to expand beyond the current biological perspective to include the concept of energy and its relationship to health (Francoeur et al. 2006).

There should be deliberation among stakeholders, administrators and practitioners on the definition of CAM and how it fulfils the agency’s mandate and vision, as well as its products and treatments. The level of medical science needed to support CAM is an important point of debate (and eventual collaboration and cohesiveness), among team members who place value on the methodology of knowledge development and evidenced based practice. Undoubtly, there will be continued demand for peer-reviewed research on the efficacy, safety and cost-effectiveness of CAM practices. (Giordano et al. 2002; Welsh et al. 2004).

Debates on the balance between an evidenced based approach and clinical expertise for clinical decision-making continue in regard to integrating CAM. This debate has the potential to either produce or reduce fears that evidenced based medicine will suppress clinical freedom and replace clinical expertise (Adams, 2000). Some CAM groups acknowledge the importance of conventional medical knowledge for a) improving their legitimacy with mainstream medicine and the public; b) safe and effective practice; and c) the move towards self regulation and professionalism. However, internal conflict exists within certain CAM groups as to the role of medical science in education, research and the legitimate use to the CAM title (Welsh et al. 2004).

Role expectations and functions and their implications for team functioning, are part of the team building activities that should occur early on in the development of an integrated health care team. Areas of team discussions should also focus on: 1) addressing attitudes and areas where there is a lack of knowledge of CAM, 2) identifying a process for coordinating conventional with CAM therapies during the treatment-recovery phase, 3) ensuring proper surveillance of patients’ health status, since CAM providers would not be expected to diagnose outside their specialty area, 4) establishing clinical expectations for reporting and accountability, 5) addressing liability concerns with respect to integrative service, 6) establishing networks with team members and internal and external service providers, and 7) establishing a referral process (Dimond, 1995; Libster, 2003; McCabe, 2005). A major barrier to the integration process involves physicians’ uncertainty and concerns regarding the legal implications of referring patients to providers who they cannot identify as qualified practitioners (McCabe, 2005). Concerns surrounding the “evidence” debate and associated liability risk will also influence referrals to CAM providers (Kelner et al. 2003). Inter-professional interactions, either through reliable referral resources or team collaboration, are fundamental to the integration of CAM into the health care system. No one individual or group of individuals will have the expertise for all possible CAM therapies (Campbell, 1998).

Professional regulatory bodies can facilitate collaboration through established professional standards or guidelines related to inter-professional boundaries. Agency policies and procedures regarding all integrative services should be based in these professional standards and clearly outline the position of the agency. Evaluation of outcomes of care should be through performance indicators reflecting an integrative approach, rather than those of one particular practitioner group, and should inform the revision of policies and practice patterns in the agency.

A major role for both practitioners and administration is in the marketing and dissemination of information on CAM natural products and services. With today’s technology, the public has access to an array of information through the internet and other public media. However, there is no control over the quality of the information that can be used by the public to inform their decisions. The agency can provide a community service by expanding awareness of CAM treatments and their potential usefulness through the dissemination of best evidence.

### Research and professional development

Networks of researchers and practitioners in integrated teams can validate integrative care through evaluative research programs. Integrated clinics need to nurture clinical environments that establish priority areas for research; allow for mentorship between practitioners and researchers in research design and critical appraisal; encourage access to academic infrastructure, resources and funding; and encourage deliberations on methodological issues (Lewith and Holgate, 2000; Giordano et al. 2002). Research participation is an essential role expectation of practitioners since sustainability of integrative models will rest on an evaluative environment. As described by Peters (2000) “integrating different aspects of health care actually improves quality of care and delivers measurable health gain” (p 59) and this can only be accomplished through ongoing knowledge development.

There continues to be segregation between conventional and CAM providers. Even though team building strategies for enhancing quality of care are not unique to CAM providers, there is a unique twist to the trust factor in these relationships due to the lack of understanding of CAM by conventional practitioners.

Any activity that reduces bias and polarization between conventional and CAM providers further enhances the chance of successful integration of CAM services. Education is one important strategy to meet this goal. Mentorship programs that share students and expose them to the practice of both CAM and conventional providers could aid in the breakdown of professional barriers, promote tolerance and provide unique experiences for students. Wetzel, Kaptchuk, Haramati and Eisenberg, (2003) discussed initiatitives in the United States where students benefit from the understanding of allopathic and CAM practitioners’ knowledge and expertise through the exchange of rotations and externships. Workshops and educational rounds, in both academic institutions and public forums, assisted with team cohesiveness through enhanced understanding of different practice realms and discussions on appropriate referral practices. In the case of one pediatric hospital, a CAM multidisciplinary team was established that provided clinical services, education and research within the hospital. This onsite approach allowed the CAM team of experts to respond to the unique needs of consumers and practitioners as they arose, thereby addressing immediate concerns and providing practical knowledge (Highfield et al. 2005).

### Regulation and legislation

In Canada, natural products are regulated by the Federal government whereas CAM practices are regulated at the provincial level. An agency functioning within an integrative practice model would be required to maintain both federal and provincial standards and monitor the professional status of CAM groups within each province. Agency leaders should be proactive in examining whether organizations of allied professions, such as pharmacists or physicians, enable or hinder integration of desired CAM services within their agencies. This is particularly important for the dispensing of natural products within the agency. There needs to be a formal process in place to monitor formulary processes and evaluate herbal supplements for inclusion in the pharmacies associated with the agency (Meenan and Vuckovic, 2003).

For a model of integration that supports conventional practitioners performing CAM service, particular attention needs to be given to professional boundaries. When health care providers attempt to integrate complementary/alternative therapies a question is raised about what is acceptable in the “grey zone” of practice that exists in any discipline’s scope of practice. It is this grey zone of practice in which practitioners struggle to determine if new skills and knowledge are congruent with the philosophical beliefs and standards of their profession. Further, the practitioner must gain recognition as a capable provider for newly selected aspects of care, and must determine the legal and professional implications of exploring new territory (Patterson, 1999). To complicate matters, there are no medical directives to cover CAM therapies, making performance of them precarious for practitioners if they act outside the scope of their employment. The employer has the legitimate right to refuse or permit a clinician to practice CAM skills as part of any employment; therefore, negotiations around acceptable CAM practice in the agency and the education required for such practice is necessary and should be outlined in the contract.

It is also important that provincial or state regulatory bodies are involved in the process for developing the agency’s policies to legitimize CAM therapies. If there is injury, the plaintiff may show that the provider did not comply with the policies, procedures or usual practice of the agency or the usual professional standards of practice. This can result in threats to license or ability to practice if inadequate preparation for CAM resulted in poor care. Discussion as to what would constitute breach of duty for the provider practicing CAM is also essential in initial negotiations since the standard of reasonable care would need to be established before initiating services.

## Conclusion

The purpose of this paper was to outline needed resources and activities and their anticipated outcomes, for those interested in implementing integrative care in a health care agency. The intention was not to outline common activities associated with integration of professionals in general, but to discuss unique considerations when integrating professionals from different paradigms of health care. A major caveat to the logic model offered here is, regardless of what has been recorded in the literature, the strategies outlined need to be interpreted within the culture of the setting and the community.

## Figures and Tables

**Figure 1.**